# The Species Identity of the Widely Cultivated *Ganoderma*, ‘*G. lucidum*’ (Ling-zhi), in China

**DOI:** 10.1371/journal.pone.0040857

**Published:** 2012-07-20

**Authors:** Xin-Cun Wang, Rui-Jiao Xi, Yi Li, Dong-Mei Wang, Yi-Jian Yao

**Affiliations:** 1 State Key Laboratory of Mycology, Institute of Microbiology, Chinese Academy of Sciences, Beijing, China; 2 Graduate University of Chinese Academy of Sciences, Beijing, China; 3 Royal Botanic Gardens, Kew, Richmond, Surrey, United Kingdom; Université Paris-Sud, France

## Abstract

Ling-zhi, a widely cultivated fungus in China, has a long history in traditional Chinese medicine. Although the name ‘*Ganoderma lucidum*’, a species originally described from England, has been applied to the fungus, their identities are not the same. This study aims to clarify the identity of this medicinally and economically important fungus. Specimens of Ling-zhi from China (field collections and cultivated basidiomata of the Chinese ‘*G. lucidum*’), *G. lucidum* from UK and other related *Ganoderma* species, were examined both morphologically and molecularly. High variability of basidioma morphology was found in the cultivated specimens of the Chinese ‘*G. lucidum*’, while some microscopic characters were more or less consistent, i.e. short clavate cutis elements, Bovista-type ligative hyphae and strongly echinulate basidiospores. These characters were also found in the holotype of *G. sichuanense*, a species originally described from Sichuan, China, and in recent collections made in the type locality of the species, which matched the diagnostic characters in the prologue. For comparison, specimens of closely related species, *G. lucidum*, *G. multipileum*, *G. resinaceum*, *G. tropicum* and *G. weberianum*, were also examined. DNA sequences were obtained from field collections, cultivated basidiomata and living strains of the Chinese ‘*G. lucidum*’, specimens from the type locality of *G. sichuanense,* and specimens of the closely related species studied. Three-gene combined analyses (ITS+IGS+*rpb2*) were performed and the results indicated that the Chinese ‘*G. lucidum*’ shared almost identical sequences with *G. sichuanense*. Based on both morphological and molecular data, the identity of the Chinese ‘*G. lucidum*’ (Ling-zhi) is considered conspecific with *G. sichuanense*. Detailed morphological descriptions and illustrations are provided in addition to discussion of nomenclature implications.

## Introduction

Ling-zhi is a famous fungus for its medicinal values well documented in the Chinese literature which can be dated back nearly two thousand years to the *Shen Nong Materia Medica* (102–200AD [1]). It symbolises happiness, good fortune, good health and even immortality in Chinese traditional culture [2]. There are thousands of publications relating to this fungus and it is now commercially cultivated on a large scale. Isolates used in medicinal studies and the commercially cultivated strains are generally named ‘*Ganoderma lucidum*’.

Ling-zhi (‘*G. lucidum*’) has been reported by numerous studies to possess properties relating to its anti-tumour, antiviral, anti-bacterial, anti-inﬂammatory, anti-oxidant, anti-platelet aggregation, hepatoprotective, hypotensive, immuno-modulating, immunosuppressive effects [3]–[6] and, especially, the activity against HIV/AIDS, improving the quality of life of the affected in Africa in recent years [7]. Many kinds of health care products and medicines derived from this fungus are produced, traded and consumed in large quantities each year. The world trade market value of ‘*G. lucidum*’ products reached US$2.5 billion in 2003 [8]. The cultivation of Lin-zhi (‘*G. lucidum*’) has become a growth industry in China and other East-Asian countries, and is spreading to other areas of the world.

*Ganoderma lucidum* (Curtis) P. Karst. was described by Curtis [9] based on material from England and the epithet was sanctioned by Fries [10]. The fungus is widely distributed in Europe, especially in the UK. Moncalvo et al. [11], [12] revealed that the species named *G. lucidum* from both Europe and mainland China was not conspecific based on analyses of nuclear ribosome DNA regions. Based on morphological examination of collections from both China and Britain, Pelger and Yao [13] also found that there was no Chinese collection referable to true *G. lucidum*. Moreover, ‘*G. lucidum*’ collected from mainland China and tropical Asia was also separated into two lineages in molecular studies [11], [12], [14], [15]. The misidentification of the fungus in most pharmacological studies was also recognized by Wasser [16].

Recently, one of the two lineages revealed by molecular studies has been re-identified as *G. multipileum* D. Hou [17], whilst the correct identity of the most widely cultivated species of *Ganoderma* in mainland China, Japan, Korea and now spreading to other parts of the world, has not yet been determined and is still named ‘*G. lucidum*’ in many scientific articles and commercial reports. In view of the importance of the species, Hawksworth [18] proposed to conserve the name *Ganoderma lucidum* with an Asian type and introduce a new name for the European species.

The first record of *Ganoderma* from China, in modern scientific research, was made by Teng in 1934 [19] with four species and one variety. One of the species recorded was named ‘*G. lucidum*’. A further 26 species were added to the Chinese records of this group in two genera, *Ganoderma* and *Amauroderma*, nearly 30 years later in 1963 [20] and some 38 species were listed in the two genera by Tai in 1979 [21]. The name *G. lucidum*, introduced to China by Teng [19], was widely accepted by his contemporary and later Chinese mycologists [21]–[25]. The members of *Ganodermataceae* reported from China was increased to 86 species in four genera – *Ganoderma*, *Amauroderma*, *Haddowia* and *Humphreya* – in the 1980s [23]. A total of 98 species in the four genera were finally assembled by Zhao and Zhang [24] in ‘Flora Fungorum Sinicorum 18: *Ganodermataceae*’, including 58 new species based on collections from China. Of the 58 new species, 10 belong to *Amauroderma* and 48 to *Ganoderma*. Apart from 12 nonlaccate and 16 laccate species with dark brown context of the 48 new species of *Ganoderma*, 20 laccate species with light-coloured context were classified in the same section with the Chinese ‘*G. lucidum*’. Recently, Wu and Dai [25] were able to distinguish morphologically 103 Chinese species in *Ganodermataceae*, adding four more new species from China, including two in *Ganoderma* (one laccate species with dark brown context and the other nonlaccate). Additionally, one new laccate variety with light-coloured context was added by Wasser et al. [26] based on collections from northeastern China.

In total, there are 20 new laccate species and one new laccate variety with light-coloured context described from China. Among them, 13 can be distinguished from the widely cultivated Chinese ‘*G. lucidum*’ (Ling-zhi) based on basidiospore characteristics, i.e. subglobose spore shape in *G. bicharacteristicum* X.Q. Zhang and *G. kunmingense* J.D. Zhao different from ovoid shape in the latter; large spore size (up to 13.0–15.0 µm long) in *G. albomarginatum* S.C. He, *G. shandongense* J.D. Zhao & L.W. Hsu, *G. stratoideum* S.C. He and *G. xingyiense* S.C. He and small size (less than 9.0 µm long) in *G. daiqingshanense* J.D. Zhao and *G. jianfenglingense* X.L. Wu compared with medium size (9.0–11.0 µm long) in ‘*G. lucidum*’ (Ling-zhi); slightly echinulate ornamentation in *G. microsporum* R.S. Hseu, *G. multipileum*, *G. ramosissimum* J.D. Zhao, *G. tenue* J.D. Zhao et al. and *G. theaecolum* J.D. Zhao, but strongly echinulate in ‘*G. lucidum*’ (Ling-zhi) [24], [26]. There are six species, i.e. *G. atrum* J.D. Zhao et al., *G. calidophilum* J.D. Zhao et al., *G. cantarelloideum* M.H. Liu, *G. hainanense* J.D. Zhao et al., *G. mongolicum* Pilát and *G. tsugae* var. *jannieae* Wasser et al., lacking Bovista-type ligative hyphae, a well preserved character in all the collections of ‘*G. lucidum*’ (Ling-zhi), while the cutis structure in *G. rotundatum* J.D. Zhao et al. is composed of irregularly parallel or interwoven hyphae, different from hymeniodermiformic cutis in ‘*G. lucidum*’ (Ling-zhi) [24], [26]. The remaining species, *G. sichuanense* J.D. Zhao & X.Q. Zhang is morphologically similar to the Chinese ‘*G. lucidum*’ in all the above characters.

The aim of this study is to clarify the identity of the Chinese ‘*G. lucidum*’ (Ling-zhi) based on both morphological and molecular data. Detailed morphological descriptions and illustrations are presented below with discussion of nomenclature implications.

## Materials and Methods

### Ethics Statement

No specific permits were required for the described field sampling because the locations are not privately-owned or protected in any way and the field studies did not involve endangered or protected species.

### Fungal Materials

Some 113 collections, including 48 samples of commercially cultivated ‘*G. lucidum*’ (Ling-zhi) from China, 55 field collections identified as the same species, and 10 specimens of *G. sichuanense* (types, authentic material and recent collections from the type locality) were examined morphologically. In addition, five related laccate species, i.e. *G. multipileum*, *G. tropicum* (Jungh.) Bres., *G. weberianum* (Sacc.) Steyaert, *G. resinaceum* Boud. as well as the true *G. lucidum*, were selected for comparison according to published molecular phylogeny of *Ganoderma* [17], [27] and the similar results derived from analyses of cumulated molecular data (unpublished) by this group. *Ganoderma multipileum* and *G. tropicum* were the sister groups of ‘*G. lucidum*’ (Ling-zhi) in the same clade and distributed in China; and *G. resinaceum* and *G. weberianum* were the representatives of another clade which also comprised laccate species with light-coloured context. A total of 45 collections of these five species were examined and one representative collection of each species was used for molecular analyses and illustration. Extraction of genomic DNA from 118 collections and from three living strains named ‘*G. lucidum*’ and one strain of *Tomophagus colossus* (Fr.) Murrill (to serve as an outgroup) deposited in the China General Microbiological Culture Collection Center (CGMCC) was performed. Amplification of DNA fragments from 28 collections and the four living strains was successfully obtained. These collections and living strains were included in phylogenetic analyses. Sources of the specimens and strains used in this study are listed in Table 1. Voucher specimens were preserved in the Fungarium, Institute of Microbiology, Chinese Academy of Sciences (HMAS). The strains were stored at 4°C on potato dextrose agar (PDA) medium and sub-cultivated at 25°C in liquid PDA medium for 14 days to collect the mycelia for DNA extraction.

### Morphological Observations

Morphological observations mainly followed the methods described previously by Wang et al. [28]. A 5% KOH solution was used as the mounting medium. Microscopic characters were observed using a light microscope (Zeiss Axiophot). Images were captured with a Zeiss Axiocam MRc digital camera using Differential Interference Contrast (DIC) microscopy and the AxioVision Rel.4.6.3 acquisition software (Zeiss). At least 30 basidiospores of each mature specimen were measured and the basidiospore size was given both with and without the myxosporium in the species description. The Q-value (length: breadth ratios) for each spore was calculated and the mean value was used in the description.

### DNA Isolation, PCR Amplification and Sequencing

Genomic DNA from specimens and fresh fungal cultures was isolated by using the modified cetyltrimethylammonium bromide (CTAB) method as described by Jiang and Yao [29]. The ITS region, including the intervening 5.8S gene, was amplified from the total DNA using the primers ITS5 and ITS4 [30]. The primers CNL12 and 5SA-Anderson were used to amplify the IGS region [31]. For the amplification of *rpb2*, the primers bRPB2-6F and bRPB2-7.1R [32] were used. PCR amplification was carried out according to the procedures described by Wang and Yao [33]. Purified PCR products were sequenced by the cyclic reaction termination method on an ABI Prism 3730 genetic analyzer (Applied Biosystems). Each fragment was sequenced in both directions for confirmation, and the sequences of the two strands were assembled with the software ContigExpress (Vector NTI Suite 6.0, InforMax Inc.).

### Phylogenetic Analyses

Five ITS sequences from the five related laccate species with light-coloured context examined morphologically above, and one from *Tomophagus colossus* (as outgroup) were retrieved from GenBank and aligned with those sequences of the same region obtained in this study for confirmation of species identity. Sequences of all the three gene partitions obtained were compiled into a combined matrix. The sequences were aligned using Clustal X 1.81 [34] and then further manually adjusted using BioEdit 5.0.6 [35]. Maximum parsimony (MP) analyses were performed using a heuristic search in PAUP 4.0b10 for Macintosh [36], with the random addition of sequences with 1,000 replicates, tree bisection-reconnection as the branch-swapping algorithm, one tree held at each step during stepwise addition, and the MULTREES option off. Gaps were treated as missing data. Bootstrap values were calculated from 1,000 replicates, with 10 heuristic searches per replicate. Bayesian Metropolis coupled Markov chain Monte Carlo (B-MCMCMC) analyses were implemented in MrBayes 3.1.2 [37] and Modeltest 3.7 [38] was used to select the best-fit models and the parameters of DNA substitution. Bayesian analyses involved 1,000,000 generations, two independent runs with four Markov chains and sampling trees every one-hundredth generation. The average split frequencies were checked to determine optimal convergence of the chains below 0.01. A 50% majority-rule consensus tree was constructed after the exclusion of the first 25% of trees from the first stage of the run (burn-in).

## Results

### Morphological Observations

**Figure 1:**
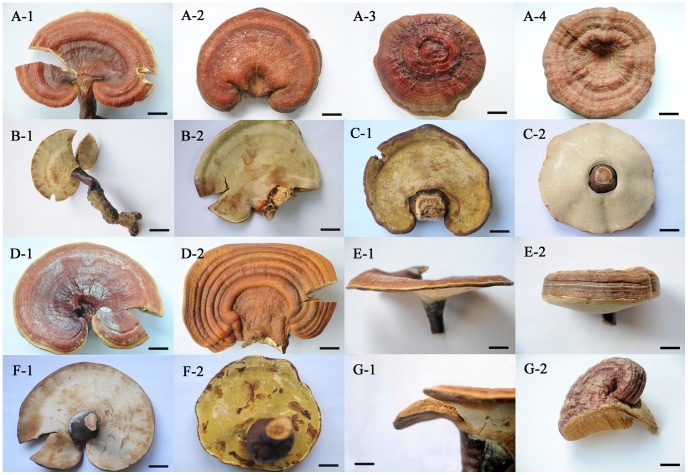
Morphological variability observed in various collections of the Chinese ‘*Ganoderma lucidum*’ (Ling-zhi). A: pileus shape; A-1: reniform and concave (HMAS 250677); A-2: reniform and convex (HMAS 240175); A-3: circular and convex (HMAS 250672); A-4: circular and concave (HMAS 240176). B: stipe length; B-1: long stipe (HMAS 250677); B-2: short stipe (HMAS 240178). C: attachment of sitpe; C-1: lateral (HMAS 240175); C-2: central (HMAS 240176). D: pileus surface; D-1: with radial furrows (HMAS 240177); D-2: with concentrically sulcate zones (HMAS 240178). E: pileus thickness; E-1: one layer and thin (HMAS 240177); E-2: several layers and thick (HMAS 240187). F: pore surface; F-1: whitish (HMAS 240177); F-2: yellowish (HMAS 250672). G: tuber length; G-1: short, less than 0.5 cm (HMAS 240177); G-2: long, more than 1 cm (HMAS 240187). Bars: 1 cm in A-1, A-3, E-2, F-2, G-1 and G-2; 1.5 cm in A-2, A-4, B-2, C-1, C-2, D-1, D-2, E-1 and F-1; 2 cm in B-1.

**Figure 2:**
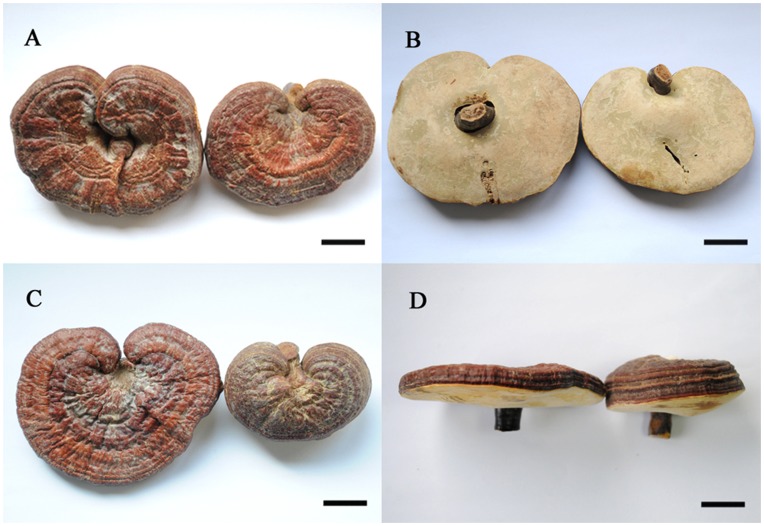
Morphological variations within a single collection, HMAS 240187. A: top view of basidiomata; B: pore surface of the same basidiomata as presented in A showing different attachment of the sitpe; C: top view of basidiomata; D: lateral view of the same basidiomata as presented in C showing different thickness of the pileus. Bars  = 1 cm.

**Figure 3:**
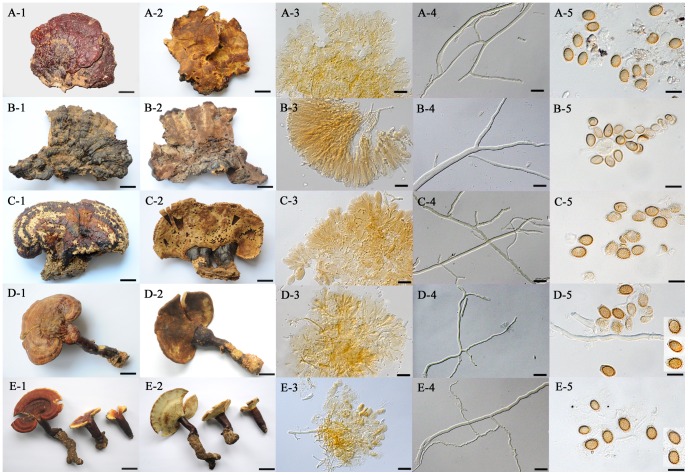
Named collections of *Ganoderma sichuanense* and one cultivated sample of Chinese ‘*G. lucidum*’ (Ling-zhi). A: HMAS 42798, holotype of *G. sichuanense*; A-1. top view of basidioma; A-2. pore surface; A-3. tissue of cutis; A-4. Bovista-type ligative hyphae; A-5. basidiospores. B: HMAS 43728, paratype of *G. sichuanense*; B-1. top view of basidioma; B-2. pore surface; B-3. tissue of cutis; B-4. skeletal hyphae; B-5. basidiospores. C: HMAS 47694, authentic specimen of *G. sichuanense*; C-1. top view of basidioma; C-2. pore surface; C-3. tissue of cutis; C-4. Bovista-type ligative hyphae; C-5. basidiospores. D: HMAS 251146, recent field collection of *G. sichuanense* from the type locality; D-1. top view of basidioma; D-2. pore surface; D-3. tissue of cutis; D-4. Bovista-type ligative hyphae; D-5. basidiospores. E: HMAS 250677, commercially cultivated ‘*G. lucidum*’; E-1. top view of basidiomata; E-2. pore surface; E-3. tissue of cutis; E-4. Bovista-type ligative hyphae; E-5. basidiospores. Bars: 2.5 cm in A-1, A-2, B-1, B-2, E-1 and E-2; 2 cm in C-1 and C-2; 1.5 cm in D-1 and D-2; 20 µm in A-3, B-3, C-3, D-3, E-3, A-4, B-4, C-4, D-4 and E-4; 10 µm in A-5, B-5, C-5, D-5 and E-5.

**Figure 4:**
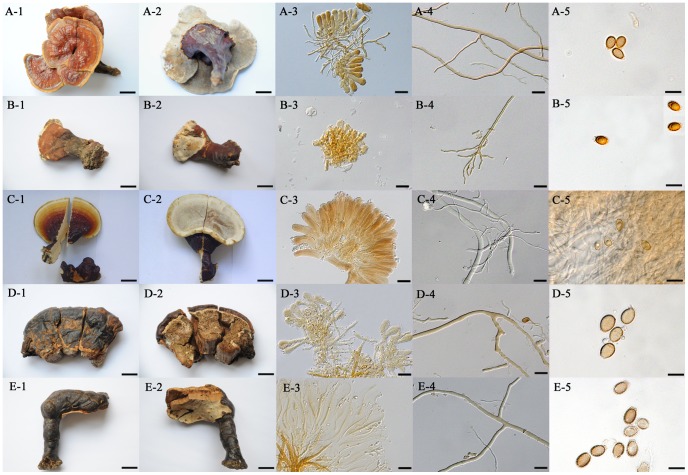
Related *Ganoderma* species with laccate pileus. A: *Ganoderma multipileum* from Sichuan, China, HMAS 242384; A-1. top view of basidioma; A-2. pore surface; A-3. tissue of cutis; A-4. Bovista-type ligative hyphae; A-5. basidiospores. B: *Ganoderma tropicum* from Hainan, China, HMAS 263143; B-1. top view of basidioma; B-2. pore surface; B-3. tissue of cutis; B-4. skeletal hyphae; B-5. basidiospores. C: *Ganoderma weberianum* from Hainan, China, HMAS 97365; C-1. top view of basidioma; C-2. pore surface; C-3. tissue of cutis; C-4. skeletal hyphae and ligative hyphae; C-5. basidiospores. D: *Ganoderma resinaceum* from U.K., HMAS 86599; D-1. top view of basidioma; D-2. pore surface; D-3. tissue of cutis; D-4. skeletal hyphae; D-5. basidiospores. E: *Ganoderma lucidum* from U.K., HMAS 86597; E-1. top view of basidioma; E-2. pore surface; E-3. tissue of cutis; E-4. skeletal hyphae; E-5. basidiospores. Bars: 2 cm in A-1, A-2, E-1 and E-2; 1 cm in B-1, B-2, D-1 and D-2; 0.5 cm in C-1 and C-2; 20 µm in A-3, B-3, C-3, D-3, E-3, A-4, B-4, C-4, D-4 and E-4; 10 µm in A-5, B-5, C-5, D-5 and E-5.

High variability was observed in macroscopic characters of the Chinese ‘*G. lucidum*’ (Ling-zhi), especially in those cultivated samples (Fig. 1). Seven characters of basidiomata were examined for their variability. The results showed that the shape of pileus varied from reniform to subcircular and also from convex to concave (Figs. 1-A-1 to 1-A-4); the length of stipe was from very short (less than the pileus radius, inconspicuous in Fig. 1-B-2) to long (more than the pileus radius, Fig. 1-B-1); attachment of the stipe to the pileus varied from lateral to nearly central (Figs. 1-C-1 and 1-C-2); the surface of pileus was either with radial furrows or with concentrically sulcate zones (Figs. 1-D-1 and 1-D-2); the thickness of pileus appeared from one layer (thinner than 1 cm) to several layers (often thicker that 1 cm; Figs. 1-E-1 and 1-E-2); the colour of pore surface varied from whitish to yellowish (Figs. 1-F-1, 1-F-2, 1-C-1 and 1-C-2); the length of tube layer was from short (less than one third of the pileus thickness) to long (more than one third of the pileus thickness; Figs. 1-G-1 and 1-G-2). The morphological variability was found not only in different specimens but also often seen in a single collection (Fig. 2). Attachment of stipe to the pileus varied from nearly central to lateral (Figs. 2-A and 2-B) and pileus thickness varied from very thin to considerably thick (Figs. 2-C and 2-D).

**Figure 5:**
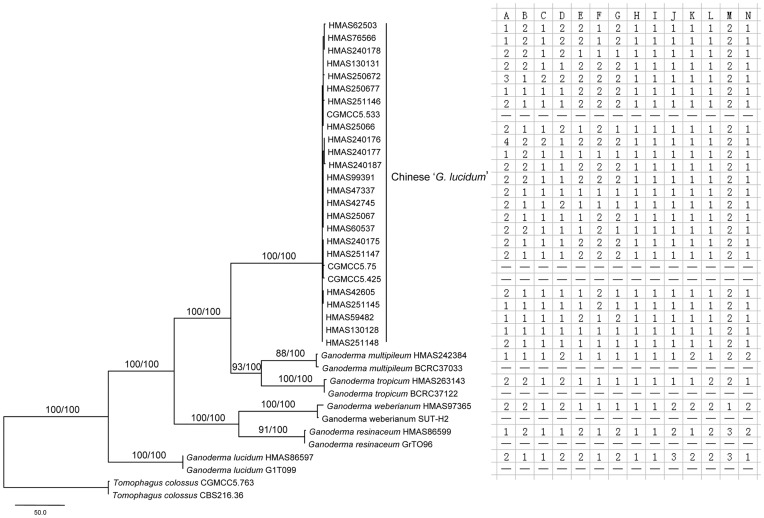
One of the 913 MP trees based on combined dataset of ITS, IGS and *rpb2*. The upper numbers on each branch denoted the percentage of bootstrap replicates/Bayesian posterior probabilities. Only bootstrap values higher than 50% in MP or 0.90 in Bayesian were shown. The letters A–G in the right table indicated macroscopic characters demonstrated in Fig. 1. A denoted the shape of pileus: 1. reniform and concave; 2. reniform and convex; 3. circular and convex; 4. circular and concave. B denoted the length of stipe: 1. long (more than the semi-diameter of pileus); 2. short (less than the semi-diameter of pileus). C denoted the attachment of sitpe: 1. lateral; 2. central. D denoted the pileus surface: 1. with radial furrows; 2. with concentrically sulcate zones. E denoted the thickness of pileus: 1. thin (<1 cm); 2. thick (>1 cm). F denoted the colour of pore surface: 1. whitish; 2. yellowish. G denoted the length of tuber: 1. short (less than one third of the pileus thickness); 2. long (more than one third of the pileus thickness). The letters H and I indicated the common characters of the six species in macroscopic morphology. H denoted the appearance of pileus: 1. laccate; 2. nonlaccate. I denoted the colour of the context: 1. light-coloured; 2. dark brown. The letters J–N denoted five microscopical characters. J denoted the length of cutis elements: 1. short (25–45 µm); 2. moderate (30–60 µm); 3. long (50–100 µm). K denoted the shape of cutis elements: 1. clavate; 2. subcylindrical. L denoted the state of Bovista-type ligative hyphae: 1. Presence; 2. Absence. M denoted the length of basidiospores cum myxosp.: 1. short (7.0–9.5 µm); 2. moderate (9.0–11.5 µm); 3. long (11.0–13.5 µm). N denoted the type of basidiospores: 1. strongly echinulate; 2. slightly echinulate.

Specimens named as *G. sichuanense*, including the holotype (HMAS 42798), paratype (HMAS 43728), an authentic specimen (HMAS 47694) and four recent collections from the type locality (HMAS 251145–251148), were examined for comparison with the cultivated Chinese ‘*G. lucidum*’ (Ling-zhi) and are shown in Fig. 3 (only HMAS 251146 was used in the figure to represent the recent field collections from the type locality). The authentic specimen and the field collection of *G. sichuanense* were stipitate while the stipes of the holotype and the paratype seemed missing or lacking (Figs. 3-A-1, 3-B-1, 3-C-1, 3-D-1). All the specimens shared the characters of radial furrows on the pileus surface (Figs. 3-A-1, 3-B-1, 3-C-1, 3-D-1), a more or less yellowish brown pore surface (Figs. 3-A-2, 3-B-2, 3-C-2, 3-D-2), and an hymeniodermiformic cutises (Figs. 3-A-3, 3-B-3, 3-C-3, 3-D-3); but the paratype specimen (HMAS 43728) was found to be different from the others in the shape of the pileus (flabelliform in the paratype, while reniform and convex in the others, Figs. 3-A-1, 3-B-1, 3-C-1, 3-D-1), the shape of cutis elements (long and subcylindrical in the paratype but short and clavate in the others, Figs. 3-A-3, 3-B-3, 3-C-3, 3-D-3), Bovista-type ligative hyphae (lacking in the paratype but present in the others, Figs. 3-A-4, 3-B-4, 3-C-4, 3-D-4), and the size and ornament of basidiospores (smaller, less than 9.5 µm long cum myxosp., and slightly echinulate in the paratype, while larger, more than 9.5 µm long cum myxosp., and strongly echinulate basidiospores in the others, Figs. 3-A-5, 3-B-5, 3-C-5, 3-D-5).

Microscopically, the cultivated Chinese ‘*G. lucidum*’ (Ling-zhi) was characterised by hymeniodermiformic cutis, short and clavate cutis elements of 20–40×7–15 µm, Bovista-type ligative hyphae and strongly echinulate basidiospores of 9.0–11.5×6.0–8.0 µm cum myxosp. and 6.5–8.5×5.0–6.5 µm sine myxosp. (Figs. 3-E-1 to 3-E-5), the same as those found in the holotype, authentic specimens and recent collections of *G. sichuanense* examined above. Microscopic differences between the cultivated samples and the paratype of *G. sichuanense* were also discovered in the hyphal system, the shape of cutis elements and the size and ornament of basidiospores (Figs. 3-E-3 to 3-E-5).

The shape of the pileus of the five related laccate species examined in this study varied from flabelliform and concave (*G. multipileum*) to flabelliform and convex (*G. tropicum*, *G. weberianum*, *G. resinaceum* and *G. lucidum*), and stipes occurred in all species except for *G. resinaceum* (Figs. 4-A-1, 4-B-1, 4-C-1, 4-E-1). The pore surface was whitish and the cutis was hymeniodermiformic in all the five species (Figs. 4-A-2, 4-B-2, 4-C-2, 4-D-2, 4-E-2), but the shape of cutis elements was different. Cutis elements were clavate in *G. tropicum* and *G. resinaceum* and subcylindrical in *G. multipileum*, *G. weberianum* and *G. lucidum*, while they were short (20–40 µm) in *G. tropicum* and *G. multipileum*, moderate (30–60 µm) in *G. weberianum* and *G. resinaceum* and very long (50–80 µm) in *G. lucidum* (Figs. 4-A-3, 4-B-3, 4-C-3, 4-D-3, 4-E-3). Bovista-type ligative hyphae occurred in *G. multipileum* but not in the other four species (Figs. 4-A-4, 4-B-4, 4-C-4, 4-D-4, 4-E-4). Basidiospores were very short (7.0–9.5 µm long cum myxosp.) in *G. weberianum*, moderate (9.0–11.5 µm long cum myxosp.) in *G. multipileum* and *G. tropicum* and long (11.0–13.5 µm ilong cum myxosp.) in *G. resinaceum* and *G. lucidum*; and slightly echinulate in *G. multipileum*, *G. weberianum* and *G. resinaceum* while strongly echinulate in *G. tropicum* and *G. lucidum* (Figs. 4-A-5, 4-B-5, 4-C-5, 4-D-5, 4-E-5).

**Table 1:** DNA sequences with their voucher materials used in this study.

Species	Voucher	Locality	ITS	IGS	*rpb2*	Reference
*Ganoderma lucidum*(Curtis) P. Karst.	HMAS 86597*	U.K.	AY884176†	**JF915427** ‡	**JF915436**	This study
	G1T099	Italy	AM269773	–	–	[55]
Chinese ‘*G. lucidum*’	HMAS 25066#	Yunnan, China	**JN197275** ‡	–	–	This study
	HMAS 25067#	Yunnan, China	**JN197276**	–	–	This study
	HMAS 42605#	Yunnan, China	**JN197277**	–	–	This study
	HMAS 42745#	Shanghai, China	**JN197278**	–	–	This study
	HMAS 47337#	Hubei, China	**JN197279**	–	–	This study
	HMAS 59482#	Henan, China	**JN197280**	–	–	This study
	HMAS 60537#	Shandong, China	**JN197281**	–	–	This study
	HMAS 62503	Jiangxi, China	**JF915405**	–	–	This study
	HMAS 76566	Shanghai, China	**JF915406**	**JF915421**	–	This study
	HMAS 99391*	Beijing, China	**JF915407**	**JF915422**	**JF915431**	This study
	HMAS 130128*	Sichuan, China	**JF915404**	–	–	This study
	HMAS 130131*	Sichuan, China	**JF915408**	–	–	This study
	HMAS 240175*	Henan, China	**JF915393**	**JF915412**	–	This study
	HMAS 240176*	Zhejiang, China	**JF915394**	**JF915413**	–	This study
	HMAS 240177*	Fujian, China	**JF915395**	**JF915414**	–	This study
	HMAS 240178*	Shandong, China	**JF915396**	**JF915415**	–	This study
	HMAS 240187*	Zhejiang, China	**JF915397**	–	–	This study
	HMAS 250672*	Henan, China	**JF915398**	–	–	This study
	HMAS 250677*	Jiangsu, China	**JF915399**	**JF915416**	–	This study
	CGMCC 5.75	China	**JN197282**	**JN197285**	**JN197288**	This study
	CGMCC 5.425	Japan	**JN197283**	**JN197286**	**JN197289**	This study
	CGMCC 5.533	Japan	**JN197284**	**JN197287**	**JN197290**	This study
*G. multipileum* D. Hou	HMAS 242384*	Sichuan, China	**JF915409**	**JF915423**	**JF915432**	This study
	BCRC 37033	Taiwan, China	EU021462	–	–	[17]
*G. resinaceum* Boud.	HMAS 86599*	U.K.	AY884177†	**JF915426**	**JF915435**	This study
	GrTO 96	Italy	AM906065	–	–	[56]
*G. sichuanense* J.D. Zhao& X.Q. Zhang	HMAS 251145*	Sichuan, China	**JF915400**	**JF915417**	–	This study
	HMAS 251146*	Sichuan, China	**JF915401**	**JF915418**	**JF915428**	This study
	HMAS 251147*	Sichuan, China	**JF915402**	**JF915419**	**JF915429**	This study
	HMAS 251148*	Sichuan, China	**JF915403**	**JF915420**	**JF915430**	This study
*G. tropicum* (Jungh.) Bres.	HMAS 263143*	Hainan, China	**JF915410**	**JF915424**	**JF915433**	This study
	BCRC 37122	Taiwan, China	EU021457	–	–	[17]
*G. weberianum* (Sacc.) Steyaert	HMAS 97365*	Hainan, China	**JF915411**	**JF915425**	**JF915434**	This study
	SUT H2	Australia	AY569451	–	–	[57]
*Tomophagus colossus* (Fr.) Murrill	CGMCC 5.763	Philippines	**JQ081068**	**JQ081069**	**JQ081070**	This study
	CBS 216.36	Philippines	Z37071Z37091	–	–	[12]

* Specimens collected by this group.

# Specimens determined by S.C. Teng or J.D. Zhao.

† Sequences submitted previously to GenBank by this group.

‡ Accession numbers in bold indicate new sequences generated from this study.

### Phylogenetic Analyses

A total of 32 ITS sequences from 28 specimens (five field collections determined as ‘*G. lucidum*’ by S.C. Teng or J.D. Zhao, 13 cultivated Chinese ‘*G. lucidum*’, five field collections of *G. sichuanense* from its type locality and five related laccate *Ganoderma* species) and four living strains (including the out group) were obtained. Attempts for DNA extraction from the holotype, paratype and authentic specimens of *G. sichuanense* failed. Several methods of DNA extraction, such as Wizard® Genomic DNA Purification Kit (Promega, U.S.A.), Chelex 100 Resin (Solarbio, China) and CTAB method, were employed for the holotype, but the specimen was found to be contaminated by an ascomycete, a species of *Eurotium*, through ITS sequencing. A basidiomycete-specific primer pair (ITS1F and ITS4B) [39] was also used to obtain ITS sequence from the holotype in PCR amplifications but no positive reaction resulted from this work. IGS sequences were successfully obtained from 20 samples, including four field collections of *G. sichuanense*, seven cultivated Chinese ‘*G. lucidum*’, five related species and four living strains. Some 13 *rpb2* sequences were obtained from three field collections of *G. sichuanense*, one cultivated Chinese ‘*G. lucidum*’, five related species and four living strains. All the sequences generated in this study were submitted to GenBank with accession numbers of JF915393–915436, JN197275–197290 and JQ081068–081070 (Table 1).

The combined 3-gene dataset with 38 taxa consisted of 2179 base pairs (ITS 561 bp, IGS 1037 bp and *rpb2* 581 bp), of which 324 were parsimony informative (ITS 85 bp, IGS 161 bp and *rpb2* 78 bp). A total of 13 taxa (one cultivated Chinese ‘*G. lucidum*’, three *G. sichuanense* field collections, five related species and four living strains) were complete for all the three genes partitions (Table 1). Maximum-parsimony (MP) analyses of the dataset yielded 947 equally parsimonious trees (length = 897, CI = 0.8016, RI = 0.7764). Tamura-Nei (TrN) model of DNA substitution with gamma-distributed rate variation across invariant sites was determined as best-fit for the combined 3-gene Bayesian analyses. Since the topology of the Bayesian consensus tree was nearly identical to that of MP analyses, one of the 947 equally parsimonious trees was presented in Fig. 5 with bootstrap values of MP analyses and posterior probabilities of Bayesian analyses.

Six major terminal clades were recognized from the combined 3-gene analyses, i.e. the Chinese ‘*G. lucidum*’ (including the field collections of *G. sichuanense*), *G. multipileum*, *G. tropicum*, *G. weberianum*, *G. resinaceum* and the true *G. lucidum* clades (Fig. 5). The five additional ITS sequences of the related laccate species and the one of *Tomophagus colossus* retrieved from GenBank clustered well with those sequences of corresponding species obtained in this study, forming the terminal clades. All the clades received support of over 85% bootstrap proportions (BP) in MP analyses and of 1.00 posterior probabilities (PP) in Bayesian analyses. *Ganoderma multipileum* and *G. tropicum* further formed a group with support of BP = 93% and PP = 1.00, sister to the lineage of the Chinese ‘*G. lucidum*’, and *G. resinaceum* and *G. weberianum* formed another group (BP = 100%, PP = 1.00).

In the Chinese ‘*G. lucidum*’ clade, a total of 26 samples included five field collections of ‘*G. lucidum*’ named by S.C. Teng or J.D. Zhao, 13 Chinese cultivated ‘*G. lucidum*’, three living strains of ‘*G. lucidum*’ and five *G. sichuanense* field collections. The clade received very strong support by both MP and Bayesian analyses (BP = 100%, PP = 1.00) and was phylogenetically separated from the true *G. lucidum* from England in the 3-gene analyses (Fig. 5).

Morphological similarities and differences of the six species of *Ganoderma* are presented in the table in Fig. 5. Letters A to G in the table represent seven macroscopic characters of basidiomata as depicted in Fig. 1, showing the high variability of the Chinese ‘*G. lucidum*’ in macroscopic morphology. It was noted that samples of the Chinese ‘*G. lucidum*’ varied in each of the seven characters. Letters H and I in the table represent macroscopic similarities of the six species in the appearance of basidiomata (laccate or nonlaccate) and the colour of the context (light-coloured or dark brown). Letters J to N indicate microscopic differences of the six species in the length and shape of cutis elements, presence of Bovista-type ligative hyphae and the length and ornament of the basidiospores.

From the table in Fig. 5, the five related laccate species of *Ganoderma* differed from the cultivated Chinese ‘*G. lucidum*’ in microscopic characters denoted by the letters J–N, i.e. *G. multipileum* in subcylindrical cutis elements (K-2) and slightly echinulate basidiospores (N-2); *G. tropicum* in the absence of Bovista-type ligative hyphae (L-2); *G. weberianum* in the longer and subcylindrical cutis elements (J-2 and K-2, 30–60 µm long), the absence of Bovista-type ligative hyphae (L-2), and the smaller and slightly echinulate basidiospores (M-1 and N-2, 7.0–9.5×5.0–7.5 µm cum myxosp.); *G. resinaceum* in the longer cutis elements (J-2, 30–60 µm long), the absence of Bovista-type ligative hyphae (L-2), and the larger and slightly echinulate basidiospores (M-3 and N-2, 11.0–13.5×7.5–9.5 µm cum myxosp.); the true *G. lucidum* from Europe in the longer and subcylindrical cutis elements (J-3 and K-2, 50–80 µm long), the absence of Bovista-type ligative hyphae (L-2) and the larger basidiospores (M-3, 11.0–13.5×8.5–10.0 µm cum myxosp.).

### Taxonomy

Based on both morphological and molecular data obtained in this study, the cultivated Chinese ‘*G. lucidum*’ (Ling-zhi) is clarified here as conspecific with *G. sichuanense*. Since the original description of *G. sichuanense* was based on very limited specimens and some published data were in contradiction with that obtained during this study, a new full description of *G. sichuanense* is provided here:

***Ganoderma sichuanense*** J.D. Zhao & X.Q. Zhang, *Acta Mycologica Sinica* 2:159. 1983. Figs. 1, 2, 3-A, 3-C, 3-D and 3-E.

**Misapplied name:** *Ganoderma lucidum sensu* S.C. Teng, *Sinensia* 5: 198. 1934.

**Basidomata** annual, stipitate, corky to woody. **Pileus** 3.0–14.5×6.0–15.5 cm, 0.5–2.5 cm thick, dimidiate, reniform to suborbicular, concave or convex; upper surface yellowish brown to bay, laccate, sometimes with radial furrows and concentrically sulcate zones; margin cream-coloured to concolourous with the pileus, acute to obtuse. **Pore** 4–6 per mm, suborbicular, rarely angular; surface whitish to yellow brown, darker when bruised; tubes up to 0.5–13 mm long, cinnamon. **Stipe** 2.0–9.0 cm long, 1.0–4.0 cm thick, lateral to eccentric, cylindrical or flattened, bay to black, laccate. **Context** 3.0–20 mm thick, cream-coloured to buff, darker near the tube layer, with or without black, horn-like layer in the context; hyphal system trimitic; generative hyphae hyaline, thin-walled, 4.0–7.0 µm diam., with clamp-connexions; skeletal hyphae near hyaline to yellow brown in 5% KOH, thick-walled to solid, aciculiform or arboriform, 3.5–9.5 µm diam.; ligative hyphae near hyaline to yellowish-brown, thick-walled, branched, 2.0–4.0 µm diam., typically Bovista-type. **Basidiospores** ovoid, truncate or not at the apex; yellowish-brown, with a dark brown eusporium bearing thick echinulae, overlaid by a hyaline myxosporium, (8.0–) 9.0–11.5 (–12.5)×(5.5–) 6.0–8.0 (–8.5) µm cum myxosp. and (5.0–) 6.5–8.5 (–9.5)×(4.5–) 5.0–6.5 (–7.0) µm sine myxosp., Q = 1.40. **Basidia** not seen. **Cutis** hymeniodermiformic, yellowish brown, elements clavate, about 20–40 µm long and 7–15 µm thick at the top.

### Specimens Examined

CHINA: ANHUI PROVINCE, Lu’an, Huoshan, cultivated, 2009, R.J. Xi & X.C. Wang SY7, HMAS 240186; Lu’an, Jinzhai, cultivated, 2009, R.J. Xi & X.C. Wang SY3, HMAS 250676; *ibid.*, SY4, HMAS 250678. BEIJING CITY, Haidian, cultivated, Jun. 1959, Z. Deng, HMAS 25103; Haidian, cultivated, *s.d.*, *s.coll.*, 141, HMAS 42780; Haidian, cultivated, 2009, R.J. Xi & X.C. Wang SY22, HMAS 240190; Huairou, cultivated from CGMCC 5.65 labeled as ‘*G. lucidum*’, Aug. 2003, X.L. Li & D.M. Wang, HMAS 99391; Mentougou, Tanzhesi Temple, Jul. 1959, G.Z. Lu, HMAS 25035; *ibid.*, 24 Aug. 1959, W.X. Wang & C.X. Pan, HMAS 25907; *ibid.*, Aug. 1960, *s.coll.*, HMAS 31634; Mentougou, Tanzhesi Temple, on ground, 25 Jul. 1958, S.C. Teng 6084, HMAS 23565; *ibid.*, on stump of *Quercus* sp., 27 Sept. 1955, S.J. Han, HMAS 17003; *ibid.*, 25 Jul. 1958, S.C. Teng 6086, HMAS 22581. FUJIAN PROVINCE, Fuzhou, cultivated, 2009, R.J. Xi & X.C. Wang SY2, HMAS 240177. GUANGXI PROVINCE, Baise, Longlin, on stump, 14 Oct. 1957, L.W. Hsu 3, HMAS 20754; Tianlin, 1997, W.Z. Ge, HMAS 77941. GUIZHOU PROVINCE, Bijie, Weining, on tree, 11 Oct. 1930, Y. Jiang 9155, HMAS 7487; Ceheng, on ground, 7 Oct. 1958, Q.Z. Wang 486, HMAS 25910; Ceheng, on rotten wood, 20 July 1985, X.L. Wu 844, HMAS 62418; Dushan, on dead tree, 19 Aug. 1930, Y. Jiang 6475, HMAS 7750; Duyun, on deak oak, 13 Jul. 1930, Y. Jiang 5825, HMAS 7510; Tongshan, on stump, 30 Jul. 1930, Y. Jiang 6121, HMAS 7513. HAINAN PROVINCE, Changjiang, Bawangling, Mar. 1978, Y.G. Meng HN1115, HMAS 37879; *ibid.*, HN1116, HMAS 37730; *ibid.*, HN1119, HMAS 37880. HEBEI PROVINCE, Shijiazhuang, Yuanshi, 15 Feb. 1975, Z.Q. Cao 132, HMAS 42534; Xingtai, Neiqiu, Sept. 1950, X.Y. Liu & F. Zhao, HMAS 15956; Zhangjiakou, Zhuolu, Mt. Dongling, 1935, *s.coll.*, 629, HMAS 15941. HENAN PROVINCE, 1989, *s. coll.*, HMAS 59482; Sanmenxia, Lushi, cultivated, 2009, R.J. Xi & X.C. Wang SY20, HMAS 240175; Zhumadian, Queshan, cultivated, 2009, R.J. Xi & X.C. Wang SY19, HMAS 250672. HUBEI PROVINCE, Wuhan, 17 Sept. 1982, *s.coll.*, HMAS 47337. HUNAN PROVINCE, *s.d.*, S.J. Han 127, HMAS 51730; Longshan, on ground, 25 Sept. 1958, L.S. Liang 900, HMAS 28128. JIANGSU PROVINCE, on oak stump, 16 Jun. 1947, C.K. Chow 1, HMAS 7515; Nanjing, Linggusi Temple, on stump, 18 Jun. 1929, S.C. Teng 412, HMAS 7511; *ibid.*, 24 May 1931, S.C. Teng 413, HMAS 7514; Nanjing, Linggusi Temple, 12 Jul. 1936, G.L. Lu 2462, HMAS 7502; *ibid.*, Sept. 1958, G.J. Lin & W.X. Wang, HMAS 23543; Nanjing, on stump in mixed broadleaf-conifer forests, 16 Jul. 1960, J.Z. Ying & H.Z. Li, HMAS 28125; *ibid.*, HMAS 28126; *ibid.*, 17 Jul. 1960, J.Z. Ying & H.Z. Li, HMAS 29745; Nantong, cultivated, 2009, X.C. Wang SY36, HMAS 250677; *ibid.*, SY37, HMAS 250679; *ibid.*, SY38, HMAS 260752; Wuxi, Yixing, 29 Aug. 1933, X.K. Deng 455, HMAS 7512. JIANGXI PROVINCE, Shangrao, cultivated, 3 Sept. 1998, J.M. Jia, HMAS 62503; Yanshan, Mt. Huanggang, 1936, X.K. Deng 17820, HMAS 16488; *ibid.*, X.K. Deng 17607, HMAS 16489. JILIN PROVINCE, Changchun, cultivated, 2009, R.J. Xi & X.C. Wang SY12, HMAS 240179; Jiaohe, cultivated, 2009, R.J. Xi & X.C. Wang SY17, HMAS 240183; *ibid.*, SY18, HMAS 240191. LIAONING PROVINCE, Dalian, cultivated, *s.d.*, F. Ji, HMAS 76763. SHANDONG PROVINCE, Heze, cultivated, *s.d.*, *s.coll.*, HMAS 59481; Laiwu, cultivated, 13 Mar. 2002, HMAS 77022; Liaocheng, Guan Xian, cultivated, 2009, R.J. Xi & X.C. Wang SY9, HMAS 240184; *ibid.*, SY10, HMAS 250675; *ibid.*, SY11, HMAS 240181; *ibid.*, SY16, HMAS 240178; *ibid.*, SY23, HMAS 240189; Qingdao, Mt. Lao, Oct. 1935, F.Y. Huang 553, HMAS 15484; *ibid.*, F.Y. Huang 555, HMAS 16294; Tai’an, 2000, D.J. Ren 1, HMAS 99390; Tai’an, cultivated, 21 Sept. 1990, X.Q. Zhang 1, HMAS 60537; Tai’an, cultivated, 21 Sept. 1990, *s.coll.*, HMAS 60542; Tai’an, cultivated, 26 Nov. 2001, N. Li, HMAS 76698; Tai’an, cultivated, 2009, X.C. Wang, SY1, HMAS 250673; Yantai, Changdao, 15 Sept. 1958, W.X. Wang, HMAS 23564. SHANGHAI CITY, cultivated, 24 Nov. 1975, *s.coll.*, 143, HMAS 42745; *ibid.*, 147, HMAS 42743; *ibid.*, 148, HMAS 42746; cultivated, 1976, *s.coll.*, 1073, HMAS 38010; Jiading, cultivated, 2009, R.J. Xi & X.C. Wang SY15, HMAS 240180; Minhang, cultivated, 28 Aug. 2001, W.L. Chen, HMAS 77009; Minhang, cultivated, 2009, R.J. Xi & X.C. Wang SY13, HMAS 240182; Xuhui, cultivated, 18 Dec. 2000, B.H. Yu, HMAS 76566. SHAANXI PROVINCE, Xi’an, cultivated, *s.d.*, X.M. Zhang, HMAS 77010. SHANXI PROVINCE, on rotten wood, 3 Aug. 1933, E. Licent, HMAS 30199. SICHUAN PROVINCE, *s.d.*, *s.coll.*, HMAS 16295; cultivated, 2003, B. Wang, Chuanzhi no.6, HMAS 99396; *ibid.*, GL8031, HMAS 130131; Chengdu, cultivated, 6 Mar. 2002, J.L. Wei, HMAS 77019; Langzhong, cultivated, 2 Mar. 2001, X.M. Zhou, HMAS 76615; Liangshan, Dechang, 14 Aug. 2003, H. Deng 347, HMAS 130128; Panzhihua, on rotten wood of broad-leaved tree, 1976, C.M. Li 116, HMAS 42798 (HOLOTYPE of *G. sichuanense* J.D. Zhao & X.Q. Zhang); Panzhihua, 2 Aug. 1982, S.C. Chen 1018, HMAS 47694; Panzhihua City, Yanbian County, Yumen Town, 2 Aug. 2010, X.C. Wang L1, HMAS 251145; *ibid.*, L6, HMAS 251146; Miyi County, Binggu Town, 5 Aug. 2010, X.C. Wang L14, HMAS 251147; *ibid.*, L21, HMAS 251148; Wanyuan, on stump of *Juglans* sp., 16 Aug. 1958, Y.N. Yu & Y.S. Xing 1002, HMAS 28127. XIZANG PROVINCE, Lhasa, cultivated, 2009, Y. Li SY34, HMAS 240185; *ibid.*, SY35, HMAS 250674. YUNNAN PROVINCE, Dali, Xiaguan, on rotten wood, 24 Aug. 1985, Q.X. Wu 570, HMAS 48243; Kunming, on stump of *Quercus* sp., 1942, W.F. Chiu, HMAS 17524; Kunming, 1979, X.H. Chen, HMAS 42605; Kunming, on ground in woods, Aug. 1989, T.S. Zhou 1072, HMAS 60538; Lijiang, Huaping, 28 May 1955, *s.coll.*, Y208, HMAS 18652; Wenshan, Guangnan, on ground, 25 Jun. 1959, Q.Z. Wang 538, HMAS 25068; Guangnan, on ground in woods, 27 Jun. 1959, Q.Z. Wang 679, HMAS 25069; *ibid.*, Q.Z. Wang 709, HMAS 27078; Guangnan, on ground in woods, 30 Jun. 1959, Q.Z. Wang 757, HMAS 26553; Qiubei, on ground in woods, 15 Jul. 1959, Q.Z. Wang 792, HMAS 25073; Qiubei, on ground in woods, 16 Jul. 1959, Q.Z. Wang 834, HMAS 25066; Qiubei, on ground in woods, 17 Jul. 1959, Q.Z. Wang 870, HMAS 25067. ZHEJIANG PROVINCE, Hangzhou, Lin’an, Mt. Tianmu, on stump, 24 Jun. 1932, S.C. Teng 1110, HMAS 7499; Jinhua, Lanxi, on base of *Acer* sp., Jul. 1977, L.G. Chen 1080, HMAS 38011; Lishui, Jingning, cultivated, 2009, R.J. Xi & X.C. Wang SY26, HMAS 240176; Qingyuan, cultivated, 2009, R.J. Xi & X.C. Wang SY32, HMAS 240187; Taizhou, Huangyan, cultivated, *s.d.*, L.F. Gan 146, HMAS 42781. VIETNAM: Nghe An, Quy Chau, Mar. 1963, *s.coll.*, 50, HMAS 32875.

### Remark

According to the original description of *G. sichuanense* [40], the size range of basidiospores was 7.4–9.5×5–7 µm cum myxosp. This range was later revised as 7.8–10.4×5.2–6.4 µm cum myxosp. [23], [24]. However, a large number of basidiospores were found in the holotype of *G. sichuanense* in this study and the size range of basidiospores was measured as 9.0–11.5×6.5–8.0 µm cum myxosp., larger than the figures given by the original authors, but not distinct from those of basidiospores found in other specimens examined here.

The small size range of basidiospores mentioned in Zhao et al. [40] might be attributed to the paratype of *G. sichuanense* (HMAS 43728), in which basidiospores were 8.0–8.5×6.0–6.5 µm cum myxosp. However, this specimen was re-determined as *G. weberianum* in this study based on microscopic characters, i.e. long and subcylindrical cutis elements (45–60×10–18 µm), colourless skeletal and ligative hyphae, absent of Bovista-type ligative hyphae, small and slightly echinulate basidiospores, and presence of chlamydospores in context.

A total of 70 specimens were identified as ‘*G. lucidum*’ by S.C. Teng [19]. Among them, 62 were preserved in HMAS but eight appear to be missing. There were also 29 specimens named as ‘*G. lucidum*’ by J.D. Zhao in HMAS. All these 91 named specimens of ‘*G. lucidum*’ were examined morphologically in this study and 63 (40 from Teng and 23 from Zhao) were re-determined as *G. sichuanense* based on the characters described here. Of the remaining 22 specimens named by Teng, five were re-determined by Zhao as *G. hainanense* (three), *G. kunmingense* (one) or *Haddowia longipes* (Lév.) Steyaert (one) and confirmed in this study, while 17 were re-determined in this study as *G. hainanense* (13), *G. mongolicum* (two) or *G. weberianum* (two). Among the remaining six specimens named by Zhao, four were re-determined here as *G. multipileum*, one as *G. hainanense* and one as *G. weberianum*. It should be noted that both S.C. Teng and J.D. Zhao applied a broad species concept to ‘*G. lucidum*’ including several *Ganoderma* species, but most of them (nearly 65% of specimens from Teng and 80% from Zhao) were *G. sichuanense*. Genomic DNA was successfully obtained from seven specimens identified as ‘*G. lucidum*’ by Teng (two) and by Zhao (five, Table 1), and their re-determination as *G. sichuanense* was further confirmed by analyses of these DNA sequences. Most of the specimens examined here were named ‘*G. lucidum*’ by various researchers and it can be concluded that *G. lucidum sensu* Chinese authors is in fact *G. sichuanense*.

## Discussion

Based on both morphological observations and molecular analyses the Chinese ‘*G. lucidum*’ (Ling-zhi) is confirmed to be distinct from the true *G. lucidum* and proved to be conspecific with *G. sichuanense*. Although the name ‘*G. lucidum*’ has been widely applied to the Chinese species of Ling-zhi in recent decades, it is a misapplied name. *Ganoderma sichuanense*, originally described from Sichuan Province in 1983, is the correct name for the widely cultivated *Ganoderma* species (Ling-zhi) even though it had not been well recognized since it was published.

The name *Ganoderma lucidum* was first introduced to the Chinese mycota by Teng [19] for collections from several provinces, i.e. Anhui, Fujian, Guangxi, Guizhou, Hainan, Jiangsu, Sichuan, Yunnan and Zhejiang. The name was then adapted by contemporary Chinese mycologists [22] and used for the *Ganoderma* species widely cultivated in China after the 1970s [21], [23]–[25], [41]. Cultivation of the species became popular in the 1980s and 1990s and the name *G. lucidum* appeared in many publications and commercial catalogs which have surged in numbers since then.

In fact, *G. lucidum* is not only incorrectly recorded in China, but is similarly reported incorrectly from around the world. The name *G. lucidum* has been applied to collections from East Africa (Ghana, Kenya and Tanzania [42]), Oceania (Australia [14]), North America (Canada and U.S.A. [15], [43]), South America (Argentina, Brazil and Uruguay [44]), South and Southeast Asia (India, Indonesia, Philippines, Thailand and Vietnam [14], [45], [46]), East Asia (China, Japan and Korea [15], [46]) as well as Europe (almost all the European countries [47]). However, the collections named as *G. lucidum* from different parts of the world have appeared in several separated lineages in phylogenetic analyses of the genus [11], [14], [15], [43], [48]. Apparently, those collections are also likely misidentified, especially if they are not from Europe, and are in need of further taxonomic investigations. One such examples is that of *G. lucidum* reported as a pathogen, causing the basal stem rot disease of oil palms (*Elaeis guineensis*) in southeastern Asia since the 1930s [49], but later studies, both morphological [50] and molecular [51], indicated that the fungus is, in fact, another species of *Ganoderma*, *G. boninense* Pat., which is further treated as a synonym of *G. orbiforme* (Fr.) Ryvarden [52].

*Ganoderma sichuanense* was described by Zhao et al. [40] based on collections from Panzhihua City (as ‘Dukou City’ before, and spelled as ‘Dokou shi’ in the protologue), Sichuan Province. In addition to the holotype (HMAS 42798), a paratype (HMAS 43728) was also cited in the protologue. The species was diagnosed as having ‘a distinctly and radially rugose pileus, with the upper surface verrucose and tuberculose; pore surface yellowish when young, becoming brown or black when bruised; and small spores distinguished from other *Ganoderma* species’ [24], [40]. It became obvious in this study that the original description was a mixture of *G. sichuanense* and *G. weberianum*, as represented by the paratype (HMAS 43728), especially in the small spores and smooth or slightly echinulate eusporium. Since the original publication, *G. sichuanense* has been rarely recorded in China, either with uncertainty [25], or with small basidiospores (7.5–10×5.5–7.25 µm cum myxosp. [53]), or without any supporting specimen [54]. The name *G. sichuanense* is almost a forgotten name, possibly as a result of the inaccurate description. Morphological observations of the holotype of *G. sichuanense* and an authentic specimens of topotype (HMAS 47694) revealed that the species was the same as the widely cultivated Chinese ‘*G. lucidum*’ (Ling-zhi), and this identity was further confirmed by the molecular evidence from recent collections (HMAS 251145–251148) from the type locality sharing primary morphological characters with the holotype.

Although the doubt on the identity of the Chinese ‘*G. lucidum*’ (Ling-zhi) was expressed by some researchers, e.g. Moncalvol et al. [11], [12], Pegler and Yao [13], and Wasser [16], the misapplication of this name had not been corrected. The proposal to conserve the name *G. lucidum* for the Chinese species [18] could be an option, but this would involve synonymising *G. sichuanense* with the former and introducing a new name for the well recognised European species. The misapplication of *G. lucidum* to the Chinese species has a relatively short history, although it has become dominant in the last few decades since the successful cultivation and intensive medicinal exploitation of the species. As stated above, *G. lucidum* has been misapplied to many different species of *Ganoderma* around the world, and conservation of this name for Ling-zhi will not provide a universal solution. The name ‘*G. lucidum*’ as used for the Chinese species is erroneous and should be corrected; *G. sichuanense* is the correct name to use.
